# Incidentally Detected Gallbladder Adenocarcinoma Presenting as Acute Calculous Cholecystitis: A Case Report and Management Considerations

**DOI:** 10.7759/cureus.104903

**Published:** 2026-03-09

**Authors:** Anupam Gupta, Felipe Alberto Camacho Cordovez, Oscar A Vazquez, Mridul Pansari

**Affiliations:** 1 Surgery, Bangalore Medical College, Bangalore, IND; 2 Surgery, Kempegowda Institute of Medical Sciences, Bangalore, IND; 3 Pathology, Jackson Memorial Hospital, Miami, USA; 4 General Surgery, University of Florida, Gainesville, USA; 5 Hepato-Pancreato-Biliary Surgery, Bayhealth Hospital, Dover, USA

**Keywords:** acute calculous cholecystitis, biliary tract malignancy, cholecystectomy, gallbladder adenocarcinoma, gallstones, hepatobiliary surgery, incidental gallbladder carcinoma, laparoscopic cholecystectomy, perimuscular invasion, radical cholecystectomy

## Abstract

Gallbladder cancer is an aggressive malignancy of the biliary tract with a poor prognosis and variable geographic distribution. It may be identified incidentally after a cholecystectomy performed for presumed benign disease, with diagnosis established on final histopathological examination. Surgeons should be aware of this possibility, as incidental detection has important implications for staging and subsequent management. Early recognition and appropriate evaluation are essential to guide treatment and improve outcomes.

## Introduction

Gallbladder cancer is the fifth most common malignancy of the gastrointestinal tract and can present early or at a very advanced stage with poor prognosis [1]. Incidentally detected gallbladder cancer refers to cancer identified in the gallbladder when cholecystectomy is performed for a reason other than cancer resection [2]. Acute calculous cholecystitis, an inflammatory condition of the gallbladder caused by gallstones, is one of the most common diagnoses for emergency cholecystectomy. Patients with acute cholecystitis often undergo emergency cholecystectomy with prior imaging findings not suggestive of malignancy [2,3].

A significant portion of gallbladder cancers are detected on cholecystectomy during histological evaluation, and subsequent staging is required based on the depth of invasion, nodal metastasis, and distant metastasis [1-3]. As chemotherapy is generally noncurative and prognosis is best with an R0 resection (complete surgical removal of the tumor with negative microscopic margins), the general surgeon needs to understand the key aspects of management of incidentally detected gallbladder cancer [4]. Distinguishing acute inflammatory gallbladder disease from an underlying malignancy is clinically important, particularly in high-risk populations, because delayed recognition may affect staging, referral to hepatobiliary specialists, and the need for additional oncologic surgery. The purpose of this report is not only to demonstrate the presence of incidentally detected gallbladder cancer but also to provide important information that needs to be communicated to the patient regarding further management when faced with a cancer diagnosis.

## Case presentation

A 74-year-old Hispanic woman from Chile with no significant past medical history presented with right upper quadrant abdominal pain, colicky in nature, with tenderness suggestive of biliary colic. Pertinent positive findings on laboratory evaluation included leukocytosis and deranged liver enzymes, as detailed in Table 1.

**Table 1:** Laboratory findings on presentation

Parameters	Patient Value	Reference Range
White blood cell count (×10³/µL)	13.2	4.0–10.5
Total bilirubin (mg/dL)	3.5	0.2–1.3
Alkaline phosphatase (U/L)	256	38–126
Aspartate aminotransferase (AST) (U/L)	1465	<55
Alanine aminotransferase (ALT) (U/L)	1503	<35

Ultrasound evaluation revealed gallstones with a prominent edematous gallbladder wall and a bile duct measuring 10 mm. On hospital day 1, her total bilirubin increased to 3.7 mg/dL, and she underwent endoscopic retrograde cholangiopancreatography with sphincterotomy and removal of stones from the bile duct, leading to improvement in liver function tests.

In view of the nonvisualization of the cystic duct, signs of gallbladder inflammation on ultrasound, and right upper quadrant pain suggestive of acute calculous cholecystitis, the patient subsequently underwent laparoscopic cholecystectomy. The procedure was performed using a four-port technique, with one port in the umbilicus and three additional ports in the epigastric, right upper quadrant, and right flank regions.

**Figure 1:**
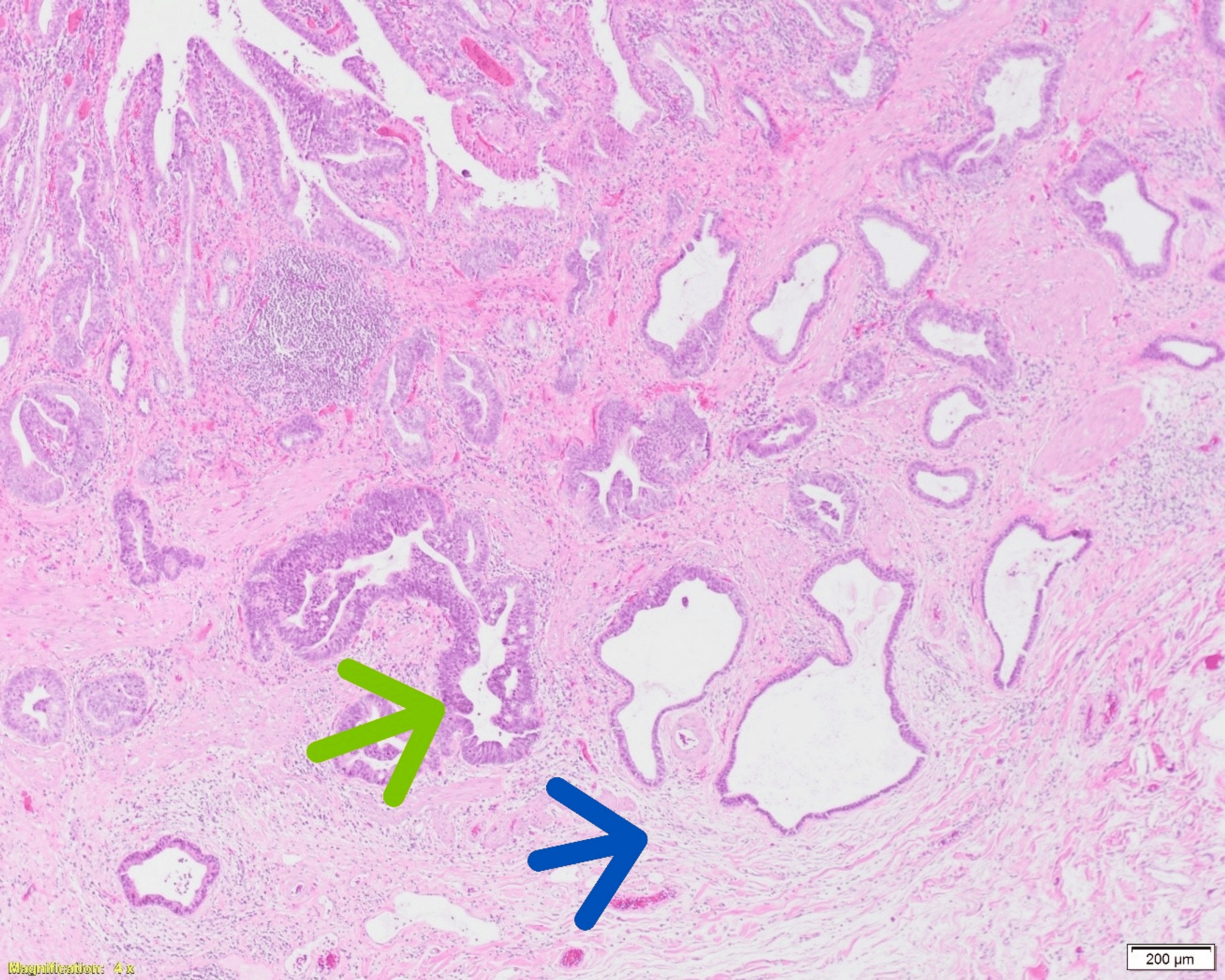
Hematoxylin and eosin (H&E) stain (4× magnification) demonstrating invasive low-grade adenocarcinoma of the gallbladder. The tumor glands (green arrow) infiltrate the perimuscular connective tissue (blue arrow), consistent with T2 gallbladder carcinoma.

Intraoperative findings included dense inflammatory adhesions with a difficult Calot’s triangle. There was a spillage of gallbladder contents into the abdominal cavity. The gallbladder was removed using an Endo-Catch bag and sent for pathological evaluation, which revealed negative cystic duct and liver bed margins and low-grade adenocarcinoma invading the perimuscular connective tissue, consistent with T2 disease (Figure 1).

Postoperative staging imaging was planned to evaluate for residual or metastatic disease, and the patient was referred to a hepatobiliary surgery team for further evaluation and management.

## Discussion

Incidentally detected gallbladder cancer is usually discovered histologically after a laparoscopic cholecystectomy performed for presumed benign gallbladder disease. This occurs in approximately 0.2%-2.9% of patients undergoing cholecystectomy and represents about 25%-40% of all gallbladder cancers identified [5].

Risk factors for gallbladder cancer include gallstones, polyps, and chronic inflammation, and it is more common in women above the age of 60. Genetic factors, obesity, chronic infection, anomalous pancreaticobiliary junction, and polyps can predispose to the development of gallbladder cancer [6]. Gallbladder cancer is more prevalent in Chile and other South American countries such as Argentina, Ecuador, and Peru, as well as in India and Eastern Europe [6].

Patients commonly present with biliary colic or acute calculous cholecystitis, leading to emergency surgical management. Imaging and clinical findings usually suggest acute inflammation rather than malignancy, with minimally invasive cholecystectomy (laparoscopic or robotic) being the standard treatment. As suspicion for cancer is low, open cholecystectomy is rarely indicated [5-7].

Preoperative imaging findings such as a polyp larger than 10 mm or asymmetrical wall thickening may suggest gallbladder cancer; however, these findings are often indistinguishable from acute inflammation. In high-risk patients, frozen section can be performed to assess the depth of invasion, and if a higher T stage is identified, radical surgery can be performed in centers with appropriate expertise. In centers without such expertise or when suspicion is low, patients should be referred postoperatively to a multidisciplinary hepatobiliary team [8,9].

An inflamed gallbladder with intraoperative spillage is a known risk factor for peritoneal dissemination and recurrence [10].

Pathological evaluation is essential to determine the depth of invasion and the margin status for further management. Gallbladder cancer staging is based on tumor depth (T), nodal involvement (N), and distant metastasis (M). Adenocarcinoma is the most common histologic type of incidentally detected gallbladder cancer. Squamous cell carcinoma represents a smaller subset and is known to behave more aggressively [11].

For early tumors involving the mucosa and lamina propria (T1a), with negative margins, cholecystectomy alone is sufficient. T1b disease is defined as a tumor invading the muscle layer. For T2 disease, involving the perimuscular connective tissue, radical cholecystectomy with resection of liver segments 4B and 5 (or 2-3 cm wedge resection of the gallbladder bed) along with regional lymphadenectomy is recommended to achieve an R0 margin [12].

From a pathological standpoint, incidental gallbladder neoplasia is easy to miss because early dysplastic or carcinomatous lesions may appear grossly normal. Careful examination is necessary when abnormalities such as wall thickening, induration, mucosal irregularity, or polyps are observed. Histologic findings of intestinal metaplasia or low-grade dysplasia warrant further section sampling to exclude advanced lesions. When high-grade dysplasia (carcinoma in situ) or invasive carcinoma is identified, more comprehensive sampling is required. Complete submission of the gallbladder is recommended for pTis, pT1, or microscopic pT2 disease, while for grossly visible pT2 lesions, four to six additional representative sections are advised [13].

Attention should be directed toward the cystic duct margin and tumor location, as tumors involving the liver side have a worse prognosis than those on the peritoneal side. Residual disease is seen in approximately 40%-80% of patients undergoing radical cholecystectomy for T2 disease. The goal of surgery is to achieve negative margins and adequate lymphadenectomy, with a minimum of six lymph nodes, including cystic and portal nodes [13,14].

The timing of radical cholecystectomy is usually four to eight weeks after the initial surgery; however, survival benefit is more dependent on staging than on timing. Surgery with negative margins remains the mainstay of curative treatment. For positive margins or advanced disease, chemotherapy with platinum-based agents has shown benefit [13-15].

## Conclusions

Incidentally detected gallbladder cancer can clinically and radiologically mimic acute calculous cholecystitis. Surgeons in the West should maintain a high index of suspicion in patients from endemic regions such as Chile. Preoperative diagnosis is challenging, and final diagnosis relies on histopathology, which assesses tumor depth, nodal status, and metastasis. It is important to note that acute inflammation can cause glandular atypia that mimics dysplasia, especially low-grade dysplasia. In such cases, careful morphologic assessment, additional sampling, and selective immunohistochemistry are critical for accurate diagnosis. Patients with T2 disease or higher require additional surgical intervention. General surgeons must recognize the potential for incidentally detected gallbladder cancer and ensure appropriate referral to hepatobiliary specialists while effectively communicating staging, prognosis, and management timing to patients.
